# Comprehensive Set of Tertiary Complex Structures and Palmitic Acid Binding Provide Molecular Insights into Ligand Design for RXR Isoforms

**DOI:** 10.3390/ijms21228457

**Published:** 2020-11-11

**Authors:** Apirat Chaikuad, Julius Pollinger, Michael Rühl, Xiaomin Ni, Whitney Kilu, Jan Heering, Daniel Merk

**Affiliations:** 1 Institute of Pharmaceutical Chemistry, Goethe University Frankfurt, Max-von-Laue-Str. 9, 60438 Frankfurt, Germany; pollinger@pharmchem.uni-frankfurt.de (J.P.); ruehl@pharmchem.uni-frankfurt.de (M.R.); ni@em.uni-frankfurt.de (X.N.); kilu@pharmchem.uni-frankfurt.de (W.K.); 2 Structural Genomics Consortium, BMLS, Goethe-University Frankfurt, Max-von-Laue-Str. 15, 60438 Frankfurt, Germany; 3 Fraunhofer Institute for Molecular Biology and Applied Ecology IME, Branch for Translational Medicine and Pharmacology TMP, Theodor-Stern-Kai 7, 60596 Frankfurt, Germany; Jan.Heering@ime.fraunhofer.de

**Keywords:** nuclear receptor, retinoid X receptor, stearic acid, neurodegeneration

## Abstract

The retinoid X receptor (RXR) is a ligand-sensing transcription factor acting mainly as a universal heterodimer partner for other nuclear receptors. Despite presenting as a potential therapeutic target for cancer and neurodegeneration, adverse effects typically observed for RXR agonists, likely due to the lack of isoform selectivity, limit chemotherapeutic application of currently available RXR ligands. The three human RXR isoforms exhibit different expression patterns; however, they share high sequence similarity, presenting a major obstacle toward the development of subtype-selective ligands. Here, we report the discovery of the saturated fatty acid, palmitic acid, as an RXR ligand and disclose a uniform set of crystal structures of all three RXR isoforms in an active conformation induced by palmitic acid. A structural comparison revealed subtle differences among the RXR subtypes. We also observed an ability of palmitic acid as well as myristic acid and stearic acid to induce recruitment of steroid receptor co-activator 1 to the RXR ligand-binding domain with low micromolar potencies. With the high, millimolar endogenous concentrations of these highly abundant lipids, our results suggest their potential involvement in RXR signaling.

## 1. Introduction

The nuclear retinoid X receptor (RXR) is a unique family of ligand-activated transcription factors, which in humans comprises of three members, namely, RXRα (NR2B1), RXRβ (NR2B2) and RXRγ (NR2B3). All three RXR isoforms typically function as a universal heterodimer partner for other nuclear receptors, including the type II thyroid hormone receptors (THRs, NR1A1-2) and the peroxisome proliferator-activated receptors (PPARs, NR1C1-3) [1,2,3]. Most of these heterodimer complexes, for example, with PPARs, are permissive and can be activated by agonists of either partner receptor, orchestrating the multifaceted regulatory roles of RXRs in gene expression. In addition, recent reports have demonstrated that RXRs can also interact with type IV nuclear receptors, such as the orphan nuclear receptor related 1 (Nurr1) [4,5]. Such heterodimers further expand the involvement of RXRs and their ligands in gene regulation. 

The RXR has been widely considered as a lipid-sensing nuclear receptor, and several lipids and fatty acids have been proposed as RXR modulators. This includes the vitamin A metabolites, 9-*cis*-retinoic acid and 9-*cis*-13,14-dihydroretinoic acid, which have been suggested as major endogenous ligands capable of activating all RXR subtypes with nanomolar potency (EC_50_ of ~0.1 µM) [6,7,8]. Naturally occurring unsaturated fatty acids, including linoleic acid, palmitoleic acid, docosahexaenoic acid and arachidonic acid, have also been proposed to act as natural RXR agonists, albeit with lower potencies in a micromolar range (EC_50_ of 5–10 µM) [9]. Considering their natural endogenous concentrations, physiological modes of action of these lipids and fatty acids on RXR activation remain sparse [10]. In addition, it remains unclear whether other types of lipids or fatty acids, such as saturated forms, could bind and activate the nuclear receptor.

Several lines of evidence have demonstrated the involvement of RXR in neurodegenerative diseases. For example, RXR expression increased after CNS injury and RXR activation counteracted CNS inflammation [11,12]. RXRγ was particularly found to be involved in the regulation of CNS myelination [13,14], a key process in multiple sclerosis with high relevance also in Alzheimer’s disease (AD). The beneficial effects of RXR modulation have been demonstrated in rodent models of multiple sclerosis [15,16] and AD [17,18,19]. This suggests RXR as a potential chemotherapeutic target for diverse neurodegenerative diseases [10,13,20,21,22,23,24], further exemplified by promising outcomes from a small clinical trial demonstrating benefits of the RXR agonist, bexarotene, in lowering the severity of AD [25]. In addition, RXRs hold a strong promise as a target for cancer treatment, and the pan-RXR agonist, bexarotene, has been approved for a limited second-line indication in a few cancer types. Nevertheless, some severe adverse effects [10,26,27] currently limit the application of RXR modulators for chemotherapeutic purposes, which is in part due to the lack of subtype selectivity. 

The human genome contains three RXR isoforms, but each subtype exhibits different expression patterns in different cells or tissues. RXRα is mainly expressed in liver, kidney and spleen, while RXRβ is ubiquitously found and RXRγ expression is mostly limited to the CNS and muscles [1,28]. All three RXR isoforms share high sequence identity and their ligand-binding pockets are predicted to be identical, presenting thereby an enormous hurdle for the development of subtype-selective ligands [29]. It has been proposed that potential subtle changes in local conformations or within the second sphere of the binding site may provide a basis for designing small molecules with subtype selectivity [29]. Although a few subtype-preferential RXR ligands have been discovered unprecedentedly through screening campaigns or by systematic structure–activity relationship studies [30,31,32], a rationale to guide subtype-selective RXR ligand design is needed. 

To date, only the ligand-bound crystal structures of RXRα and RXRβ have been reported with most studies focused on the alpha isoform. Here, we report a complete set of the crystal structures for all three RXR isoforms in active, ligand-bound conformation with an identical ligand. These tertiary complexes provide complete structural insight into the active state of all RXRs. A structural comparison indeed demonstrated high conservation, yet subtle differences within the second sphere of the binding pockets were observed. In addition, we discovered saturated fatty acids, including palmitic acid (PA), myristic acid (MA) and stearic acid (SA), as RXR modulators capable of inducing the recruitment of co-regulators to the RXR ligand-binding domain (LBD) and hence, activating the receptor. The flexible nature of the saturated fatty acids enabled an L-shape conformation compatible for filling the vast hydrophobic cavity of the RXR ligand-binding pockets. Collectively, our discovery of this new ligand type and the comprehensive structural information provide important insights into RXR modulation and a fundamental basis for future RXR ligand design. 

## 2. Results

### 2.1. Unprecedented Identification of Palmitic Acid Binding to RXRα

**Figure 1:**
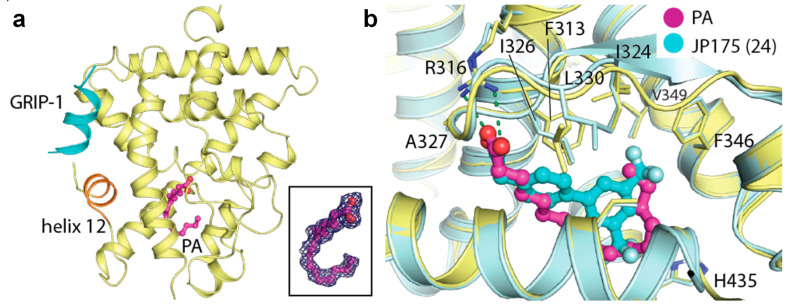
Palmitic acid binding to retinoid X receptor α (RXRα). (**a**) Overview of the crystal structure of the tertiary complex between RXRα, palmitic acid (PA; magenta sticks) and glucocorticoid receptor interacting protein 1 (GRIP-1) activating peptide (blue). Inset shows |F_O_|-|F_C_| omitted electron density map contoured at 3σ for the bound fatty acid. (**b**) Superimposition of the JP175- and PA-complexed RXRα structures reveals that PA occupies the same space in the binding pocket as the small molecule modulator with no structural alteration observed upon its binding.

Upon crystallization and structure determination of the recombinant RXRα LBD in complex with the glucocorticoid receptor interacting protein 1 (GRIP-1) co-regulator peptide, we unexpectedly observed extra electron density within the ligand-binding pocket of the protein. Based on the shape of the density, we hypothesized the presence of a fatty acid, likely contaminated from the recombinant protein production in *Escherichia coli*. Mass spectrometric analysis further confirmed the identity of this ligand as palmitic acid (PA), a widely abundant fatty acid in bacteria and eukaryotic cells, which was successfully modelled in the crystal structure (Figure 1a). A structural comparison with other known RXRα ligands, such as JP175 (compound 24) [31], revealed that PA occupied the orthosteric binding pocket typically targeted by other small molecule modulators [10,33] and the accommodation of this fatty acid did not require any structural alterations (see Section 2.3) (Figure 1b). 

The unprecedented discovery of PA binding to RXRα is rather intriguing. Saturated fatty acids have not been described as RXR ligands to date. Thus, this RXRα-PA-GRIP-1 tertiary complex opens the possibility of involvement of fatty acids in RXR signaling. Of note, the similarity between the binding mode of PA and that of potent synthetic agonists [34] suggests an ability of saturated fatty acids to induce RXR activation (see Section 2.5).

### 2.2. Palmitic Acid Also Interacted with Other RXR Isoforms

**Figure 2:**
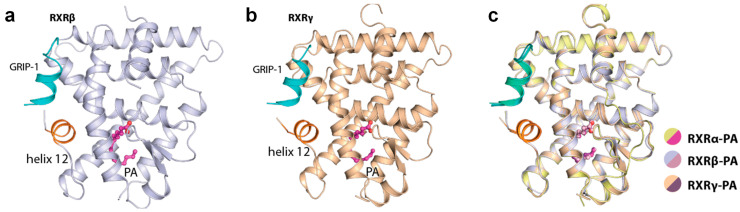
Palmitic acid (PA) bound to all RXR isoforms and induced similar active conformations. Overview of the tertiary complexes between PA, GRIP-1 co-regulator peptide and RXRβ (**a**) and RXRγ (**b**). (**c**) Superimposition of RXRα, RXRβ and RXRγ structures reveals highly similar active conformation in this tertiary complex with PA and the peptide.

We next questioned whether PA could also bind to the other two RXR isoforms and therefore, we crystallized and successfully determined the tertiary complex structures of both RXRβ and RXRγ with (exogenously added) PA and the GRIP-1 co-regulator peptide. The crystal structures indeed revealed the binding of PA to both isoforms in a similar manner to that observed for the alpha counterpart (Figure 2a,b). These structures provided to our knowledge not only the first report of the ligand-bound RXRγ crystal structure, but a complete set of the same tertiary complexes of all three RXRs with an identical ligand. Overall, binding of PA induced similarly an active state of RXRα, RXRβ and RXRγ. The three structures were highly identical, supported by the superimposition root-mean-square deviation of atomic positions (RMSD) of 0.29 Å (Figure 2c). This suggested similar active conformations shared across the RXR receptor family.

### 2.3. Binding of PA in RXRs

**Figure 3:**
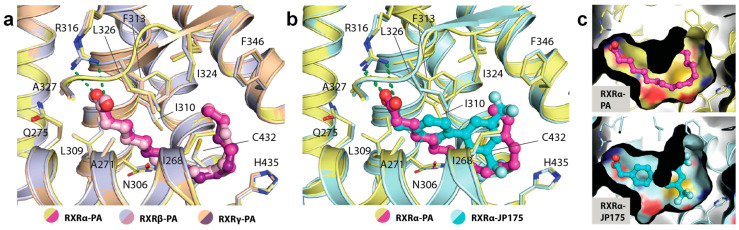
The binding mode of palmitic acid in RXRs. (**a**) Detailed interactions between PA and three RXR isoforms demonstrate high conservation of the binding mode of the fatty acid. Numbering is based on RXRα. (**b**) Structural comparison reveals PA occupying a similar space as typically exploited by other synthetic small molecule modulators. (**c**) Surface representation comparing the binding modes of PA and JP175 [31] suggests that highly flexible PA with L-shape conformation potentially utilizes a space-filling mechanism for its binding to RXRs.

A detailed structural analysis showed that PA occupied identically the canonical ligand-binding site and engaged in similar interactions in all three RXR isoforms. No conformational changes were observed for the residues lining the pocket (Figure 3a). The carboxylic acid moiety of the fatty acid formed a salt-bridge contact with the highly conserved Arg316 (using the numbering of RXRα as an example), a canonical interaction typically observed for most RXR ligands [10]. The saturated hydrocarbon tail was seen to elongate along the hydrophobic groove until C11-C12 where an ~90° bend was formed to ascend the rest of the tail toward the hydrophobic pocket created by Phe346 and Leu324. In comparison with RXR synthetic small molecule modulators, such as JP175 (compound 24) [31], this L-shape conformation allowed the fatty acid to fully occupy the vast hydrophobic space of the ligand-binding pocket without the need for structural rearrangement of the lining residues (Figure 3b,c). Such induced binding conformation compatible for the binding pocket is likely enabled by the flexible nature of PA.

### 2.4. Structural Analysis of Amino Acid Differences among RXR Isoforms

The complete set of comparable active state structures enabled the comparison of the LBDs of RXRα, RXRβ and RXRγ. Sequence analysis shows that the three isoforms share high sequence identity (~82–86%). We observed that none of those amino acid differences were lining the ligand-binding pocket, confirming previous assumptions that the RXR ligand-binding sites are conserved. In addition, the interface of the receptors involved in GRIP-1 co-regulator peptide binding was also highly identical. 

**Figure 4:**
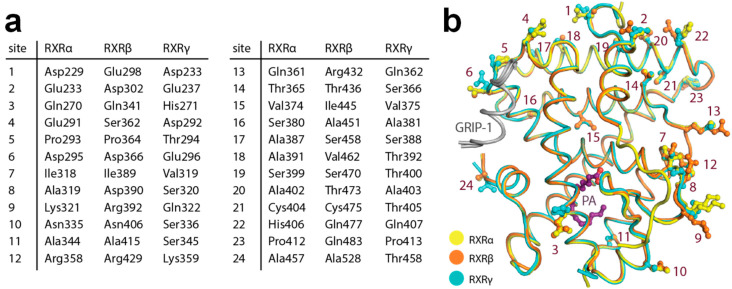
Amino acid differences within the ligand-binding domain (LBD) of the three RXR isoforms. (**a**) Summary of the residue differences. (**b**) Mapping of the substitutions onto the RXR structure with the numbering scheme according to (**a**).

To understand whether the 24 amino acid differences within the LBD region may have any indirect influence toward ligand-binding properties among the three isoforms, we mapped the differences onto the structures (Figure 4a,b). We observed that although most changes were located peripherally at the surface of the proteins, there were three substitutions in the proximity of the ligand-binding pocket (site 3, site 11 and site 15 based on Figure 4a). Among them, a minor difference in the lipophilic residue at site 15 (α: Val374, β: Ile445, γ: Val375) located in helix 8 might affect interactions between helices 5 and 8. Since helix 5 is involved in ligand-binding site formation, this difference could allow contributions to subtype preference through differential ability of helix 5 movement in the RXR isoforms. Additionally, the amino acid change at site 3 with polar, aliphatic glutamine in the alpha and beta isoforms (Gln270 and Gln341, respectively) to basic, aromatic histidine (His271) in the gamma counterpart was particularly notable. This substitution could result in alterations of the protein properties, such as electrostatic charge, evident, for example, by a contact between helix 2 His271 and helix 12 Asp449 observed in the RXRγ structure, which was absent for the glutamine residues at the equivalent position in the other two isoforms. Such changes within the second sphere of the binding site might potentially influence different structural stabilization, essentially for helix 2 that constructs the binding pocket, and thus might indirectly affect the integrity and properties of the ligand-binding pockets.

### 2.5. PA Acts as a Modulator of RXRs

**Figure 5:**
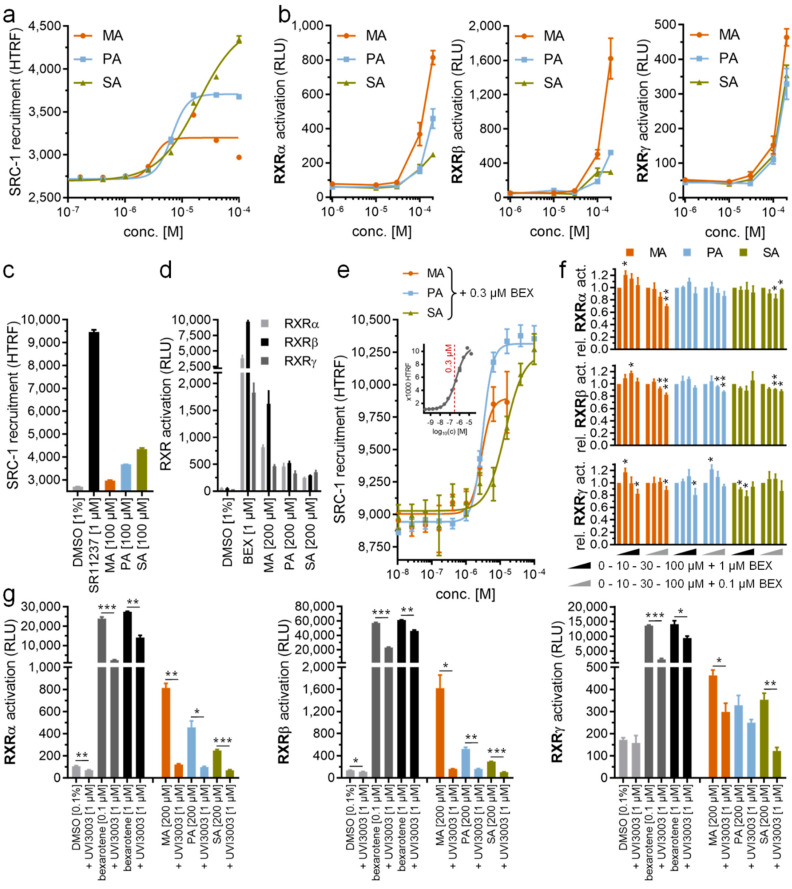
In vitro characterization of saturated fatty acids as RXR ligands. (**a**) Myristic acid (MA), palmitic acid (PA) and stearic acid (SA) dose-dependently induced steroid receptor co-activator 1 (SRC-1) recruitment to the RXRα LBD in a HTRF-based assay with low micromolar potencies. (**b**) MA, PA and SA activated RXRα, RXRβ and RXRγ in cellular reporter gene assays at 100 µM concentration and above. (**c**,**d**) Efficacy of saturated fatty acids in promoting SRC-1 recruitment to the RXRα LBD (c) and in activating RXR in a cellular setting (d) was low compared with the synthetic agonists, SR11237 and bexarotene. (**e**) In the presence of 0.3 µM bexarotene (~EC_50_), MA, PA and SA showed no competitive behavior, but caused enhanced SRC-1 recruitment at higher concentrations. Inset shows dose response of bexarotene in the same setting with the 0.3 µM concentration used for competition experiments highlighted in red. (**f**) At a low concentration (10 µM), saturated fatty acids showed a tendency to promote bexarotene (0.1 µM or 1 µM)-induced RXR activation in Gal4 hybrid reporter gene assays too, while weak competitive behavior was observed at a higher concentration (100 µM). (**g**) RXR activation by MA, PA and SA (200 µM each) in the cellular reporter gene assay was blocked by the RXR antagonist, UVI3003. All data are mean ± SEM values, *n* = 3. * *p* < 0.05, ** *p* < 0.01, *** *p* < 0.001 (*t*-test vs. bexarotene alone (**f**) or as indicated (**g**))

The tertiary complex structures showed that all PA-bound RXRα, RXRβ and RXRγ adopted an active state [34], suggesting that binding of PA induces conformational changes necessary for binding a co-activator. To examine whether PA can act as a modulator that activates RXRs in solution, we next performed homogenous time-resolved fluorescence resonance energy transfer (HTRF)-based co-regulator recruitment assays using a peptide derived from steroid receptor co-activator 1 (SRC-1) as established previously [35]. In addition to PA, we also tested the RXR modulator potentials of myristic acid (MA; C14:0; C_14_H_28_O_2_; C_13_H_28_COOH) and stearic acid (SA; C18:0; C_18_H_36_O_2_; C_17_H_36_COOH). We observed indeed that all three saturated fatty acids promoted SRC-1 recruitment to RXRα, with an increase in their efficacies seemingly corresponding to longer chain lengths (EC_50_ of 3.2 µM for MA, 6.6 µM for PA and 19 µM for SA) (Figure 5a). Nevertheless, their activities in the low micromolar range were inferior to that of synthetic RXR agonists, such as our positive control SR11237 (Figure 5c). 

In Gal4 hybrid reporter gene assays, MA, PA and SA activated RXRα, RXRβ and RXRγ at 100 µM concentrations and above (Figure 5b) with equally moderate efficacies (Figure 5d) as observed in the cell-free setting. The weaker activity in cellular assays could likely be due to rapid processing of the fatty acids by cells in conjugation, energy generation or membrane integration, thus explaining the lack of previous reports on RXR activation by saturated fatty acids [9]. Additionally, MA, PA and SA might bind to other fatty acid-sensing proteins [36] in the cellular setting which would additionally reduce available concentrations for RXR activation in this assay. Nonetheless, the abilities of saturated fatty acids to activate RXR in the cell free recruitment assay and the cellular reporter gene assays open a possibility of MA, PA and SA, typically present in high abundance at both endogenous and intracellular levels, as potential physiologically relevant RXR agonists.

Since we observed partial RXR agonism for MA, PA and SA, we also studied whether the fatty acids showed competitive behavior with the RXR agonist, bexarotene. Thus, we titrated MA, PA and SA in the presence of 0.3 µM bexarotene corresponding to approximately the compound’s EC_50_ in this assay (Figure 5e). We observed no competition of the fatty acids with bexarotene, but in contrast detected an increase in HTRF at high fatty acid concentrations indicative of enhanced RXR activation. The EC_50_ values of MA, PA and SA in this setting were congruent with their activity in the absence of bexarotene (EC_50_ of 2.9 µM for MA, 3.1 µM for PA and 14 µM for SA in the presence of 0.3 µM bexarotene). These additive effects of the fatty acids and bexarotene are likely due to incomplete occupation of the RXR LBD by bexarotene at its EC_50_ concentration, allowing further activation by other agonists. In the cellular reporter gene assays, MA, PA and SA at low concentrations (10 µM) also exhibited a tendency to further promote RXR activation in the presence of bexarotene (0.1 µM or 1 µM), while a weak competitive behavior with bexarotene (0.1 µM or 1 µM) was observed at higher concentrations (100 µM) despite not reaching statistical significance for all fatty acids on all RXR isoforms (Figure 5f). Although non-specific effects in the cellular setting may partly affect the results in this competitive experiment, the RXR agonistic effect of MA, PA and SA at 200 µM was fully reversed by the RXR antagonist, UVI3003 [37], at 1 µM (Figure 5g), confirming their activities as directly mediated by RXR. 

## 3. Discussion

The nuclear RXRs, which play a key role in transcriptional regulation, are appealing molecular targets for the treatment of neurodegenerative diseases and cancer. However, adverse effects currently limit further exploration and exploitation of their chemotherapeutic potential. Heterodimer- and subtype-specific targeting has been proposed as a strategy to overcome this, yet high sequence similarity between the three human RXR isoforms presents a challenge for the design of such selective modulators. Several examples demonstrate that RXR ligands can exhibit heterodimer preference (e.g., for RXR:PPAR [31,38,39,40], RXR:LXR [41] and RXR:Nurr1 [42]) and even subtype preference [30,31] but while some structural insights in heterodimer-preference mechanisms are available, subtype preference among RXRs remains elusive. As a basis for better understanding of the three RXR isoforms and potential selectivity mechanisms, we presented here a uniform set of the crystal structures for all three RXR isoform LBDs in activated conformation, providing improved structural insights for the design of RXR modulators. The comparative structural analysis revealed that the binding pockets of all three RXR isoforms are highly identical, yet subtle differences at the second sphere residues, such as those at helix 8 and 2, are evident. These amino acid substitutions could control the integrity of the pocket, and thus indirectly affect the ligand-binding properties. This notion was in line with previous structure–activity relationship studies on biphenyl-based RXR agonists [31], where an introduction of bulkier moieties at the terminal ring or substituents at the ortho position of the phenylacetic acid motif binding in the vicinity of helix 5, which forms an interface with helix 8, was observed to result in different affinities to the three RXR isoforms. In addition, various RXR-ligand complexes, including RXRα-LG100754 (PDB ID: 6sti) [43], RXRα-JP175 (PDB ID: 6sjm) [31], RXRα-honokiol derivative (PDB ID: 5mku) [42], RXRβ-LG100268 (PDB ID: 1h9u) [44] and RXRγ-PA, have demonstrated different conformations of helix 12 and the loop preceding this helix, feasibly required for accommodation of different ligands. Since this helix and the loop form a direct interface with helix 2, the small change from Gln270/341 in RXRα/β to His271 in RXRγ, leading to a hydrogen bond with helix 12 Asp449 in the latter, could result in a different degree of flexibility of this region, which may have an indirect effect toward the properties of the ligand-binding pockets. However, a ligand with distinct preference toward particular subtypes as well as structural insights into the binding of which will be needed to further validate rational targeting of these features for selective ligand design. Nevertheless, achieving such a scenario could remain a challenge.

With the discovery of saturated fatty acids as RXR ligands, we made an unprecedented finding with potential relevance to RXR physiology and pharmacology. As seen from the crystal structures, SRC-1 co-regulator recruitment and RXR activation in reporter gene assays, PA, MA and SA were discovered as novel RXR modulators that activate RXR at micromolar concentrations. Such an observation may have important implications for understanding endogenous RXR activation. Of note, the endogenous concentrations of PA and related saturated fatty acids reach millimolar levels in plasma and are likely higher in intracellular compartments. In a systematic study on plasma lipid levels in young healthy Canadian adults [45], PA was the most abundant fatty acid with a maximum concentration as high as 4.0 mM, which is a remarkable three orders of magnitude above the lipid’s EC_50_ value for SRC-1 recruitment to the RXR LBD. SA and MA reached plasma levels up to 1.0 mM and 0.3 mM, respectively. The previously known natural unsaturated fatty acid RXR ligands, such as arachidonic acid (maximum plasma concentration, 0.8 mM [45]; EC_50_ (RXRα), ~10 µM [9]) and docosahexaenoic acid (maximum plasma concentration, 0.2 mM [45]; EC_50_ (RXRα), ~10 µM [9]), are less abundant. In the cellular reporter gene assays, the potency of MA, PA and SA regarding RXR activation was lower than in the cell-free setting, which is likely due to their cellular metabolic processing or storage leading to markedly reduced free concentrations and may be a reason why RXR agonism of saturated fatty acids has not been observed previously. In addition to the long-known unsaturated fatty acids as RXR agonists [9], our findings on saturated fatty acid binding to RXR expand a pool of endogenous RXR ligands. High abundance of naturally occurring saturated fatty acids raises an assumption that these lipids may considerably contribute to endogenous RXR activation that might be mainly mediated by highly abundant low-affinity ligands.

## 4. Materials and Methods 

### 4.1. Expression and Purification of RXR Isoform LBDs

The LBDs of RXRα (NR2B1, UniProt entry: P19793, aa 223–462), RXRβ (NR2B2, UniProt entry: P28702, aa 298–533) and RXRγ (NR2B3, UniProt entry: P48443, aa 233–463) were subcloned into pNIC28-Bsa4, and the recombinant proteins were expressed in *E. coli* Rosetta strain. In brief, cells were initially cultured in TB media at 37 °C to OD_600_ of 1.6–1.8, prior to cooling down to 18 °C. At OD_600_ of 2.6–2.8, isopropyl-β-D-1-thiogalactopyranoside (IPTG) at 0.5 mM was added to induce protein expression overnight. Cells were harvested, resuspended in buffer (50 mM Tris, pH 7.5, 500 mM NaCl, 5 mM imidazole, 5% glycerol and 1 mM tris(2-carboxyethyl)phosphine (TCEP)) and lysed by sonication. Recombinant proteins were purified by Ni-affinity chromatography. The N-terminal histidine tag was removed by treatment with Tobacco Etch Virus (TEV) protease, and the cleaved proteins were further purified by reverse Ni-affinity chromatography. Size-exclusion chromatography was performed and the proteins were stored in a buffer containing 20 mM Tris, pH 7.5 and 150 mM NaCl.

### 4.2. Crysallization and Structure Determination

Crystallization of the recombinant RXR LBDs (10–13 mg/mL) was performed using the sitting drop vapor diffusion method at 20 °C and conditions listed in Table 1. Crystals were cryoprotected with mother liquor supplemented with 20% ethylene glycol. Diffraction data were collected at DESY P13 and SLS X06SA, and were processed and scaled using XDS [46] and Aimless [47], respectively. The initial structure solution was achieved based on the coordinates of RXRα (PDB ID: 6sjm) by molecular replacement using Phaser [48], followed by alternating model rebuilding and structure refinement in COOT [49] and Refmac [50], respectively. The final models were verified for their geometric correctness using Molprobity [51]. Data collection and refinement statistics are summarized in Table 1. 

### 4.3. Hybrid Reporter Gene Assays for RXRα, RXRβ and RXRγ

The Gal4 hybrid reporter gene assays for RXRα, RXRβ and RXRγ were performed as described previously [53,54] using the Gal4-fusion receptor plasmids pFA-CMV-hRXRα-LBD (NR2B1, UniProt entry: P19793, aa 225–462), pFA-CMV-hRXRβ-LBD (NR2B2, UniProt entry: P28702-1, aa 294–533) and pFA-CMV-hRXRγ-LBD (NR2B3, UniProt entry: P48443-1, aa 229–463), which code for the hinge region and LBD of the canonical RXR isoform. pFR-Luc (Stratagene, San Diego, CA, USA) as the reporter plasmid and pRL-SV40 (Promega, Madison, WI, USA) as the internal control were used. HEK293T cells were grown in DMEM high-glucose medium (supplemented with 10% fetal calf serum (FCS), sodium pyruvate (1 mM), penicillin (100 U/mL) and streptomycin (100 μg/mL)) at 37 °C and 5% CO_2_, and seeded in 96-well plates (3.0 × 10^4^ cells/well) on the day before transfection. Right before transfection, medium was changed to Opti-MEM without supplements and cells were transiently transfected using the Lipofectamine LTX reagent (Invitrogen, Carlsbad, CA, USA) according to the manufacturer’s protocol with pFR-Luc (Stratagene), pRL-SV40 (Promega) and the respective pFA-CMV-hRXR-LBD clone. After 5 h of incubation, medium was changed to Opti-MEM (supplemented with penicillin (100 U/mL) and streptomycin (100 μg/mL)) additionally containing 0.1% DMSO and the respective test compounds or 0.1% DMSO alone as the untreated control. After overnight incubation (12–14 h), cells were assayed for luciferase activity using the Dual-Glo luciferase assay system (Promega) according to the manufacturer’s protocol on a Tecan Spark M10 luminometer (Tecan Deutschland GmbH, Crailsheim, Germany). Each sample was tested in at least three biologically independent repeats in duplicates. Data were normalized for transfection efficiency and cell growth by division of firefly luciferase data by Renilla luciferase data and multiplying by 1000 to obtain relative light units (RLUs). Fold activation denotes the mean RLU of test compounds at a respective concentration divided by the mean RLU of untreated control. Bexarotene was used for assay validation and served as the synthetic reference RXR agonist.

### 4.4. SRC-1 Recruitment Assay (HTRF)

The recruitment of the SRC-1 co-activator-derived peptide to the RXRα LBD (aa 225–462) was studied in a homogeneous time-resolved fluorescence (HTRF) resonance energy transfer-based system. Terbium cryptate as streptavidin conjugate (Cisbio Bioassays, Codolet, France) was used as the fluorescence resonance energy transfer (FRET) donor and stably coupled via an N-terminal biotin to the SRC-1-derived peptide (biotin-CPSSHSSLTERHKILHRLLQEGSPS) (Eurogentec Deutschland GmbH, Cologne, Germany), which contains the co-activator consensus motif, LxxLL. Recombinant RXRα LBD fused to N-terminal superfolder green fluorescent protein (sGFP) served as the FRET acceptor. Coactivator recruitment by the RXRα LBD brings FRET donor and acceptor into close proximity, resulting in a gain in the FRET signal as indicator for binding. The assay was performed in HEPES buffer (25 mM HEPES, pH 7.5 adjusted with KOH, 150 mM KF, 5% (*w/v*) glycerol, 0.1% (*w/v*) CHAPS and 5 mM 1,4-dithiothreitol (DTT)). Assay solutions were prepared in this buffer containing 12 nM recombinant sGFP-RXRα LBD, 12 nM SRC-1-FRET donor complex and 1% DMSO with test compounds at varying concentrations or DMSO alone. Fluorescence intensities (FI) at 520 nm (acceptor) and 620 nm (donor reference) after excitation at 340 nm were recorded on a Tecan Spark luminometer equipped with enhanced fluorescence module (Tecan) after 2 h of incubation at room temperature. To obtain dimensionless HTRF, FI520 nm was divided by FI620 nm and multiplied with 10,000. The recruitment of the SRC-1-derived peptide to the RXRα LBD was validated with increasing concentrations of SR11237.

## Figures and Tables

**Table 1:** Data collection and refinement statistics.

Complex ^a^	RXRα-PA-GRIP-1	RXRβ-PA-GRIP-1	RXRγ-PA-GRIP-1
PDB codes	7A77	7A78	7A79
Beamline	DESY P13	SLS X06SA	SLS X06SA
*Data Collection*			
Resolution ^b^ (Å)	43.10–1.50 (1.55–1.50)	45.0–1.72 (1.78–1.72)	46.01–2.05 (2.12–2.05)
Space group	*P* 4_3_2_1_2	*P* 4_3_2_1_2	*P* 2_1_2_1_2_1_
Cell dimensions	a=b=66.2, c=110.6 Å	a=b=63.6, c=110.1 Å	a=63.3, b=67.1, c=110.7 Å
	α=β=γ=90.0	α=β=γ=90.0	α=β=γ=90.0°
Number of unique reflections	40,177 (3855)	24,821 (2373)	30,235 (2910)
Completeness (%)	100.0 (100.0)	100.0 (100.0)	99.9 (100.0)
I/σI	20.0 (4.0)	15.3 (2.3)	8.2 (2.0)
R_merge_ (%)	0.073 (0.703)	0.059 (0.875)	0.148 (0.782)
CC (1/2)	0.999 (0.876)	0.999 (0.769)	0.994 (0.754)
Redundancy	15.6 (14.2)	8.0 (8.3)	8.5 (7.9)
*Refinement*			
Number atoms in refinement (P/G/L/O) ^c^	1841/ 99/ 18/ 237	1688/ 111/ 18/ 160	3416/ 210/ 36/ 182
B factor (P/G/L/O) ^c^ (Å^2^)	22/ 29/ 29/ 35	37/ 40/ 39/ 44	38/ 48/ 47/ 43
R_fact_ (%)	15.2	17.7	19.5
R_free_ (%)	18.0	20.4	24.8
rms deviation bond ^c^ (Å)	0.015	0.013	0.014
rms deviation angle ^c^ (°)	1.6	1.4	1.4
*Molprobity Ramachandran*			
Favor (%)	97.4	99.0	98.0
Outlier (%)	0	0	0
Crystallization condition ^d^	17% PEG 3350, 0.2 M ammonium acetate, 0.1 M tris, pH 8.0	16% low-molecular-weight PEG smears, 0.1 M HEPES, pH 7.5, 5% ethylene glycol, 0.1 M KCl	17% PEG 3350, 0.1 M potassium citrate

^a^ GRIP-1 peptide sequence is KHKILHRLLQDSSY. ^b^ Value in brackets indicates high-resolution shell statistics. ^c^ P/G/L/O indicates protein, GRIP-1 peptide, palmitic acid ligand and others; rms indicates root-mean-squre. ^d^ Low-molecular-weight PEG smears contain PEG 400, PEG 550 MME, PEG 600 and PEG 1000 [52].

## Abbreviations

AD	Alzheimer’s disease
DTT	dithiotreitol
FI	fluorescence intensity
FRET	fluorescence resonance energy transfer
GRIP-1	glucocorticoid receptor-interacting protein-1
HTRF	homogenous time-resolved fluorescence resonance energy transfer
LBD	Ligand-binding domain
MA	myristic acid
Nurr1	nuclear receptor related 1
PA	palmitic acid
PPAR	peroxisome proliferator-activated receptor (NR1C1-3)
RMSD	root-mean-square deviation of atomic positions
RXR	retinoid X receptor (RXRα: NR2B1; RXRβ: NR2B2; RXRγ: NR2B3)
SA	stearic acid
SRC-1	steroid receptor co-activator 1
THR	thyroid hormone receptor (NR1A1-2)
